# Positive rate and risk factors of latent tuberculosis infection among persons living with HIV in Jiangsu Province, China

**DOI:** 10.3389/fcimb.2023.1051060

**Published:** 2023-03-07

**Authors:** Yu Zhang, Peng Lu, Kai Wu, Hongxi Zhou, Haibing Yu, Ju Yuan, Lang Dong, Qiao Liu, Wei Lu, Haitao Yang, Dianyi Cao, Limei Zhu

**Affiliations:** ^1^ Department of Chronic Communicable Disease, Center for Disease Control and Prevention of Jiangsu Province, Nanjing, Jiangsu, China; ^2^ School of Public Health, Southeast University, Nanjing, Jiangsu, China; ^3^ Jiangsu Prison Administration, Central Hospital, Changzhou, Jiangsu, China; ^4^ Health Policy Research Department, Jiangsu Provincial Health Development Research Center, Jiangsu, China

**Keywords:** latent tuberculosis infection, risk factors, positive rate, HIV, prison

## Abstract

**Objective:**

To analyze the latent tuberculosis infection (LTBI) among persons living with HIV(PLWH) in Jiangsu Province, to explore the factors affecting the positive rate of LTBI, and to take appropriate measures to control tuberculosis (TB) infection.

**Methods:**

A cross-sectional study was conducted among PLWH in Jiangsu Province from June to July 2021. All PLWH in Jiangsu Province were used as the study population. Currently, the diagnosis of LTBI lacks a “gold standard” and can only be assisted by the immunological method. In this study, Tuberculin skin test (TST), ESAT6-CFP10 test (EC), and QuantiFERON-TB gold in-tube (QFT) were used to detect the positive rate of LTBI among PLWH and to analyze their risk factors.

**Results:**

A total of 340 prisoners were included, 89.7% were male, the median age was 38 years [Interquartile Range (IQR):32-46 years], these patients were on Antiviral Therapy (ART), and median CD4 counts was 376 (IQR: 261-496), 103 (30.3%) were positive in at least one test, LTBI by TST was 16.5%, LTBI by EC was 15.9%, LTBI by QFT was 26.2%. Univariate analysis showed the results for TST, EC, and QFT were not affected by CD4 counts (p>0.05), and multivariate analysis showed that a history of incarceration was associated with an increased risk of positive TST (adjusted odds ratio [aOR]=1.98;95% CI,1.03-3.82), EC (aOR=2.65;95% CI,1.37-5.12) and QFT (aOR=2.01;95%CI,1.12-3.57), in addition, female gender was associated with increased risk of positive TST (aOR=3.66;95%CI,1.60-8.37) and EC (aOR=3.43;95%CI,1.46-8.07), and contact history of TB patients was associated with increased risk of TST (aOR= 2.54;95%CI,1.23-5.22) and QFT (aOR=2.03;95%CI,1.03-3.99), and ethnic minorities (aOR=0.26;95%CI,0.12-0.57), longer duration of incarceration was associated with an increased risk of positive QFT (aOR=1.12;95%CI,1.02-1.24). Conclusions Female gender, and ethnic minorities, history of incarceration, longer duration of incarceration, and contact history of TB patients are risk factors for LTBI among PLWH in Jiangsu Province, and attention should be paid to TB control in this population.

## Introduction

Tuberculosis (TB) was a chronic infectious disease caused by Mycobacterium tuberculosis (MTB). According to the Global TB Report 2021, there were an estimated 9.87 million new TB cases globally and an estimated 1.28 million deaths from TB-related diseases (WHO, 2021a). There are about 842,000 new cases of TB (WHO, 2021a) in China in 2021, ranking second among the 30 countries with a high burden of TB (Liu et al., 2022b), second only to India. Although the incidence of TB has been declining in recent years, the rate of decline was slow and far from reaching the goal of “End TB” (WHO, 2015). To achieve this goal sooner, attention should be paid to the screening and treatment of people with latent tuberculosis infection(LTBI) (WHO, 2015). LTBI was a persistent immune response to the antigenic stimulation of MTB without any clinical signs of active TB (Liu et al., 2013). There were 20% of people infected with MTB in China (Lu et al., 2021), which was one of the countries with the highest burden of LTBI in the world (Lu et al., 2022). And people with LTBI were 5%-10% likely to develop active TB in their lifetime (Vynnycky and Fine, 1997). World Health Organization (WHO) guidelines suggested that importance should be attached to TB control among people at high risk of LTBI (including persons living with HIV (PLWH) and prisoners) (WHO, 2021b). WHO consolidated guidelines on tuberculosis Module 2: Screening Systematic screening for tuberculosis disease showed that PLWH were approximately 19 times more likely to develop TB than HIV-uninfected people (WHO, 2021b). The incidence of disease and mortality of TB among prisoners was higher than that of the general population, and the incidence of TB among prisoners is estimated to be 23 times higher than the general population (Baussano et al., 2010). PLWH had dual risk factors for latent TB infection. A meta-analysis that included 22 papers showed that the positive rate of TB among PLWH was 32.6% (Dianatinasab et al., 2018), higher than that of the general population. HIV would increase the risk of latent TB infection in PLWH before incarceration (Baillargeon et al., 2004), and risk factors such as crowded living conditions, psychological stress, and limited ventilation environment after incarceration further increased the risk of LTBI in prisoners (Baillargeon et al., 2004). TB among PLWH not only transmitted among prisoners, but also to their family and community members through prisoners, prison staff, and visitors. Therefore, TB control in the general population may be affected (Stuckler et al., 2008; Barbour et al., 2010). TB/HIV dual infection has a low treatment success rate and high mortality rate (Yang et al., 2016; Maimaiti et al., 2017; Liu et al., 2022a), and ensuring early detection and treatment is the key to reducing morbidity and mortality. In recent years, a new test, the EC skin test, has been developed and licensed in China, through the immunological reaction to detect LTBI. Several clinical trials (Li et al., 2016; Xu et al., 2021) in China have shown that EC is sensitive and specific and that the test is a low-cost, simple procedure that reduces the cost of LTBI screening and epidemiological investigations. There are no studies on EC in PLWH, but this study aims to conduct a study on EC, IGRA, and TST in PLWH at the same time, to describe the prevalence and factors influencing positive results for each of the three tests.

## Materials and methods

### Study design and population

This study was a cross-sectional study conducted in a prison hospital in Jiangsu Province from June to July 2021. All PLWH were included in our study. After obtaining their informed consent, all the subjects were tested using three methods, including tuberculin skin test (TST), ESAT6-CFP10 test (EC), and QuantiFERON-TB gold in-tube (QFT), then they were surveyed by questionnaire. Blood samples and questionnaires were collected, and the information was checked to eliminate unqualified samples.

### Inclusion criteria

(1) Patients without clinical manifestations of active TB and abnormalities on chest X-ray examination; (2) Patients who were willing to participate in the study and signed the informed consent form;

### Exclusion criteria

Patients who did not meet the inclusion criteria.

### Experimental operation

The blood of all subjects was first collected for QFT.TB detection, and then the in-body two-arm skin test was performed with EC recombinant fusion antigen and TB-PPD. Before starting the skin test, skin test reagents, resuscitation drugs, and resuscitation equipment should be prepared to prevent the occurrence of serious adverse events, and after injecting EC in the right forearm and observing no abnormalities for 5 minutes, then inject PPD in the left forearm. All participants were observed for acute adverse reactions for 30 minutes. If there were no abnormal reactions, they could leave on their own and return 48-72 hours later for observation of infusion or erythema. This was performed by trained medical workers, who measured the longitudinal and transverse diameters of infusion with a vernier caliper (Li et al., 2016).

### Judgement of results

TST: the reaction at the injection site should be examined at 48-72 hours after injection, with local subcutaneous induration as the criterion. Measurements should be recorded by mm of the transverse and longitudinal diameter of induration. In PLWH, the average induration diameter is no less than 5mm as a positive reaction, and local blister, necrosis, and lymphangitis are all strong positive reactions. EC (Xu et al., 2021): The injection site reaction should be examined 24-72 hours after injection, with local subcutaneous induration or redness as the standard. The number of mm of transverse diameter and longitudinal diameter of induration or erythema was measured and recorded respectively, the one with the largest induration or erythema was the criterion, and the average reaction diameter [transverse diameter + longitudinal diameter)/2] ≥5mm was considered a positive reaction. All those who have a blister, necrotic, or lymphangitis belong to a strong positive reaction. QFT (Kussen et al., 2016): (1) Positive: results ≥0.35IU/mL and ≥25% negative control, and negative control ≤8.0IU/mL; (2) Negative: results of <0.35IU/ml or <25% negative control, negative control ≤8.0IU/mL and positive control ≥0.5IU/mL; (3) If one of the following two conditions is met, the result is uncertain: results <0.35 IU/ml or <25% negative control with a negative control ≤8.0 IU/ml and a positive control <0.5 IU/ml; Negative control >8.0 IU/ml.

### Questionnaire development

A self-made questionnaire was used to formulate the preliminary questionnaire according to the TB epidemiological case questionnaire, and the final questionnaire was determined by combining expert consultation and group discussion. After the professional training of investigators, with the informed consent of the subjects, a questionnaire survey was conducted to investigate each object again, which included age, gender, ethnicity, height, weight, education level (receiving education above primary school level), employment (before the current incarceration), income(before the current incarceration), and history of incarceration, duration of incarceration, smoking status(before the current incarceration), contact history with TB patients, the presence of BCG scars (checked by medical personnel during blood sampling).

### Statistical analysis

Epidata3.0 was used to establish the database and input data, SPSS 26.0 software was used for statistical analysis, quantitative data were expressed by $\bar{\text{x}}$ ± S, and qualitative data were expressed by frequency(percent). T-test was used for quantitative data and Pearson’s chi-square test was used for qualitative data. Variables with P<0.05 were screened and included in the multivariate analysis. Logistic regression was used for multivariate analysis to analyze the factors influencing LTBI in PLWH.

## Results

**Figure 1:**
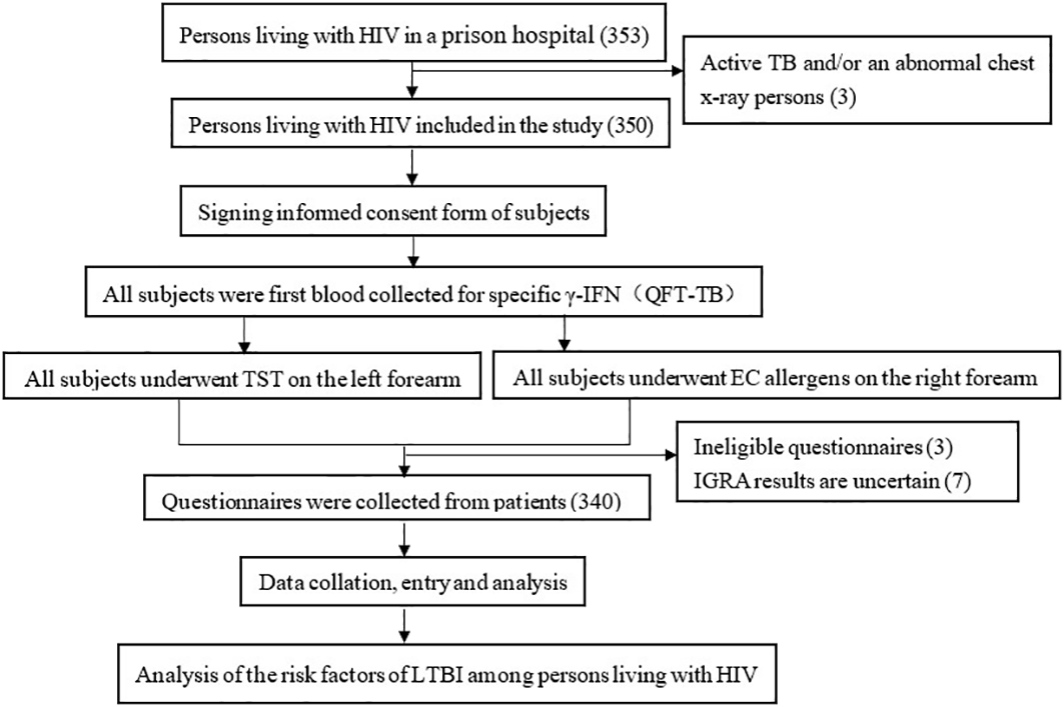
Technical routes in study of positive rate of and risk factors for LTBI among persons living with HIV, Jiangsu Province.

From June to July 2021, there were 350 PLWH in a prison hospital in Jiangsu Province, of which 340 were finally included in our study (**Figure 1**). The positive rate of latent TB infection were 16.5%, 15.9%, and 26.2% of TST, EC skin test, and QFT, respectively. 89.7% (305/340) of the study participants were male, the median age was 38 years [Interquartile Range (IQR):32-46 years], the majority 72.4% (246/340) had received an education of above primary school level), 71.8%(244/340) had no history of incarceration, 86.2%(293/340) had CD4 count > 200, 73.8% (251/340) were local population, 85.3%(290/340) had no contact history with tuberculosis patients, 34.1%(116/340) had no co-infection with other chronic diseases (Diabetes, Cardiovascular disease, Silicosis Kidney disease, Tumours, Long-term use of immunosuppressive drugs included), 54.4%(185/340) had no BCG scars, and 72.9%(248/340) had a history of smoking, 43.2% (147/340) had been incarcerated for >3 years, 41.8% (142/340) had an annual household income >20,000 RMB before incarceration, 36.2% (123/340) were unemployed before incarceration (**Table 1**).

**Table 1:** Demographic characteristics of the recruited persons living with HIV in the study.

Demographic Characteristics	All (N,%)
Gender	
Male	305 (89.7)
Female	35 (10.3)
Age,years	
≤40	205 (60.3)
>40	135 (39.7)
Ethnicity	
Han	295 (86.8)
other	45 (13.2)
Education level	
Primary School or below	94 (27.6)
Above primary School	246 (72.4)
Employment before incarceration	
No	244 (71.8)
Yes	96 (28.2)
Income before incarceration	
≤20000 RMB	198 (58.2)
>20000 RMB	142 (41.8)
History of incarceration	
No	123 (36.2)
Yes	217 (63.8)
Duration of incarceration,y	
≤3	193 (56.8)
>3	147 (43.2)
CD4 counts	
≤200	47 (13.8)
>200	293 (86.2)
Residence	
Local	251 (73.8)
Migrant	89 (26.2)
Contact history	
No	290 (85.3)
Yes	50 (14.7)
Concurrent condition	
No	116 (34.1)
Yes	224 (65.9)
BCG scars	
No	185 (54.4)
Yes	155 (45.6)
Smoke before incarceration	
No	92 (27.1)
Yes	248 (72.9)

(N=340).

**Table 2** shows significant factors associated with LTBI diagnosed by TST, EC, and QFT. CD4 counts were not associated with an increased risk of positive TST, EC, and QFT test results (P>0.05). History of incarceration, duration of incarceration >3 years, and annual household income ≤20,000 RMB before incarceration were not associated with an increased risk of positive TST, EC, and QFT test results (P<0.05). Furthermore, TST-positive individuals were more common in female (P=0.003), ethnic minorities (P=0.048), had a contact history with TB patients (P-0.044), history of smoking (P=0.043). Compared with EC-negative individuals, EC-positive individuals were more common in female (P=0.008), and those with no occupation before incarceration (P=0.046). Compared with QFT-negative individuals, QFT-positive individuals were more common among ethnic minorities (P=0.001), those without education (P=0.010), migrants before incarceration (P =0.031), had a contact history with TB patients (P=0.006), has a history of smoking (P=0.012).

**Table 2:** Risk factors associated with a positive LTBI diagnostic test among persons living with HIV in Jiangsu Province: univariate analysis.

Characteristic	TST		EC		QFT	
	No. (%) positive	*P*	No. (%) positive	*P*	No. (%) positive	*P*
Gender		0.003		0.008		0.119
Male	44 (14.4)		43 (14.1)		76 (24.9)	
Female	12 (34.3)		11 (31.4)		13 (37.1)	
Age,y		0.598		0.167		0.240
≤40	32 (15.6)		28 (13.7)		49 (23.9)	
>40	24 (17.8)		26 (19.3)		40 (29.6)	
Ethnicity		0.048		0.092		0.001
Han	44 (14.9)		43 (14.6)		63 (21.4)	
other	12 (26.7)		11 (24.4)		26 (57.8)	
Education level		0.410		0.722		0.010
Primary School or below	18 (19.1)		16 (17.0)		34 (36.2)	
Above primary School	38 (15.4)		38 (15.4)		55 (22.4)	
Employment before incarceration		0.081		0.046		0.081
No	26 (21.1)		26 (21.1)		39 (31.7)	
Yes	30 (13.8)		28 (12.9)		50 (23.0)	
Income before incarceration		0.002		0.049		0.002
≤20000 RMB	43 (21.7)		38 (19.2)		64 (32.3)	
>20000 RMB	13 (9.2)		16 (11.3)		25 (17.6)	
History of incarceration		0.044		0.004		0.007
No	34 (13.9)		30 (12.3)		54 (22.1)	
Yes	22 (22.9)		24 (25.0)		35 (36.5)	
Duration of incarceration,y		0.045		0.010		0.001
≤3	25 (13.0)		22 (11.4)		34 (17.6)	
>3	31 (21.1)		32 (21.8)		55 (37.4)	
CD4 counts		0.461		0.529		0.238
≤200	6 (12.8)		6 (12.8)		9 (19.1)	
>200	50 (17.1)		48 (16.4)		80 (27.3)	
Residence		0.149		0.101		0.031
Local	37 (14.7)		35 (13.9)		58 (23.1)	
Migrant	19 (21.3)		19 (21.3)		31 (34.8)	
Contact history		0.005		0.089		0.006
No	41 (14.1)		42 (14.5)		68 (23.4)	
Yes	15 (30.0)		12 (24.0)		21 (42.0)	
Concurrent condition		0.131		0.895		0.538
No	24 (20.7)		18 (15.5)		28 (24.1)	
Yes	32 (14.3)		36 (16.1)		61 (27.2)	
BCG scars		0.308		0.314		0.887
No	27 (14.6)		26 (14.1)		49 (26.5)	
Yes	29 (18.7)		28 (18.1)		40 (25.8)	
Smoke before incarceration		0.043		0.590		0.012
No	9 (9.8)		13 (14.1)		15 (16.3)	
Yes	47 (19.0)		41 (16.5)		74 (29.8)	

LTBI, latent tuberculosis infection; TST, tuberculin skin test; EC, ESAT6-CFP10 497 skin test; QFT, QuantiFERON-TB gold in-tube.

In multivariate analysis, we identified three factors independently associated with an increased risk of TST positivity, female had a higher risk of LTBI than male (adjusted odds ratio [aOR] 3.66, 95% CI 1.60-8.37), PLWH with a history of incarceration had a 1.98 (95% CI 1.03-3.82) times higher risk of LTBI than those without a history of incarceration, and PLWH with a contact history with TB patients had a 2.54 (95% CI 1.23-5.22) times greater risk of LTBI than those without a contact history with TB patients. Two factors were identified that were independently associated with an increased risk of EC positivity, with female having a higher risk of LTBI than male (aOR 3.43, 95%CI 1.46-8.07), and PLWH with a history of incarceration having a 2.65 times higher risk of LTBI than those without incarceration (95%CI 1.37-5.12). Identified four factors independently associated with an increased risk of positive QFT, with ethnic minority PLWH having a higher risk of LTBI than Han (aOR 0.26, 95% CI 0.12-0.57) and PLWH with a history of incarceration having a 2.01 (95% CI 1.12-3.57) times higher risk of LTBI than those without a history of incarceration, The risk of LTBI was 2.03 (95% CI 1.03-3.99) times higher among PLWH with a contact history of TB patients than those without. The results also showed that the longer duration of incarceration was associated with an increased positive rate of LTBI among PLWH (aOR 1.12, 95% CI 1.02-1.24) and that the longer the duration of incarceration, the higher the risk of LTBI among PLWH (**Table 3**).

**Table 3:** Risk factors associated with a positive LTBI diagnostic test among persons living with HIV in Jiangsu Province: multivariate analysis.

Characteristic	TST		EC		QFT	
	cOR (95%CI)	aOR (95%CI)	cOR (95%CI)	aOR (95%CI)	cOR (95%CI)	aOR (95%CI)
Gender						
Male						
Female	3.10 (1.44-6.67)	3.66 (1.60-8.37)	2.79 (1.28-6.11)	3.43 (1.46-8.07)	1.78 (0.86-3.71)	1.95 (0.87-4.38)
Age,y	1.17 (0.65-2.09)	0.99 (0.95-1.03)	1.51 (0.84-2.71)	0.99 (0.96-1.03)	1.34 (0.82-2.19)	1.00 (0.97-1.04)
Ethnicity						
Han						
other	2.07 (0.99-4.32)	1.52 (0.69-3.34)			3.86 (1.76-8.46)	5.04 (2.62-9.69)
Education level						
Primary School or below						
Above primary School					0.51 (0.30-0.85)	1.08 (0.56-2.06)
Employment before incarceration						
No						
Yes			0.55 (0.31-0.99)	0.81 (0.43-1.54)		
Income before incarceration	0.36 (0.19-0.71)	1.00 (1.00-1.00)	0.54 (0.29-1.00)	1.00 (1.00-1.00)	0.45 (0.27-0.76)	1.00 (1.00-1.00)
History of incarceration						
No						
Yes	1.84 (1.01-3.34)	1.98 (1.03-3.82)	2.38 (1.31-4.33)	2.65 (1.37-5.12)	2.02 (1.21-3.38)	2.00 (1.12-3.57)
Duration of incarceration,y	1.80 (1.01-3.20)	1.04 (0.93-1.16)	2.16 (1.20-3.91)	1.10 (0.99-1.22)	2.80 (1.70-4.60)	1.12 (1.02-1.24)
Residence						
Local						
Migrant					1.78 (1.05-3.01)	1.60 (0.86-2.99)
Contact history						
No						
Yes	2.60 (1.31-5.19)	2.54 (1.23-5.22)			2.36 (1.27-4.41)	2.03 (1.03-3.99)
Smoke before incarceration						
No						
Yes	2.16 (1.01-4.60)	1.70 (0.78-3.76)			2.18 (1.18-4.04)	1.54 (0.79-2.99)

LTBI, latent tuberculosis infection; TST, tuberculin skin test; EC, ESAT6-CFP10 534 skin test; QFT, QuantiFERON-TB gold in-tube; cOR, crude odds ratio; aOR, adjusted odds ratio.

## Discussion

In this study, the positive rate of LTBI by TST was 16.5%, the positive rate of LTBI by EC was 15.9%, and the positive rate of LTBI by QFT was 26.2%. Moreover, female, ethnic minorities, history of incarceration, longer duration of incarceration, and contact history with TB patients are risk factors for LTBI. The positive rate of LTBI by QFT was higher than that of TST, which was consistent with the results of most previous studies (Stephan et al., 2008; Xin et al., 2017). For example, a study carried out in German PLWH (Stephan et al., 2008)showed that the positive rate of LTBI by QFT was 20.0%, and the positive rate of LTBI by TST was 12.8%, which reflected the higher sensitivity of the QFT. There were no studies on EC in the PLWH at present, and the positive rate of LTBI detected by EC in this study was similar to that of TST and lower than that of QFT, which might indicate that the sensitivity of EC was more affected by the immune status of PLWH than QFT. The positive rate of LTBI in this study was lower than that of immunocompetent prisoners in other studies, China (QFT:52%) (Zhang et al., 2020), Ethiopia (QFT:51.2%) (Chekesa et al., 2020), and Spain (TST:54.6%) (López De Goicoechea-Saiz et al., 2018). PLWH might have false negative test results due to low immunity, thus underestimating the positive rate of LTBI in this population (Lin WC. et al., 2016; Xin et al., 2017). The positive rate of LTBI was higher in PLWH (López De Goicoechea-Saiz et al., 2018; Martinez et al., 2021), and some studies had found that the positive rate of LTBI in PLWH could be 25% higher than observed (Moura et al., 2011). In this study, CD4 count did not affect the test results of TST, EC, and QFT, which was inconsistent with the results of some studies (Lin AW. et al., 2016; Goletti et al., 2020; Keramat et al., 2020; Krutikov et al., 2022), most of which showed that TST was affected by CD4 counts, while QFT was not, probably because 86.2% (293/340) of PLWH had CD4 counts>200, test results in this population were less affected by immunosuppression, the immune response should be close to normal (Dehority et al., 2017), the proportion of CD4 counts below 100 was no more than 2%, and most people had an undetectable HIV loads, their HIV infection was well-controlled, and most of our participants had all received Antiviral Therapy(ART), previous studies (Jones et al., 2000; Badri et al., 2002; Santoro-Lopes et al., 2002; Tilahun et al., 2019) had shown that ART could improve the immune status of PLWH and reduced the incidence of TB in PLWH. There were no studies on EC skin tests in PLWH, so the relationship between EC skin tests and CD4 counts was not known.

This study showed that both histories of incarceration and longer duration of incarceration were risk factors associated with LTBI, and the positive rate of LTBI was higher among PLWH with a history of incarceration, as in other studies (Hussain et al., 2003; Margolis et al., 2013; Al-Darraji et al., 2014; Rueda et al., 2014), the longer the duration of incarceration of PLWH was associated with higher positive rate of LTBI, which was consistent with previous studies (Rueda et al., 2014; Carbone Ada et al., 2015; Aguilera et al., 2016; Chekesa et al., 2020; Zhang et al., 2020), as the longer the exposure, the higher the risk of LTBI, the positive rate of LTBI increased significantly by 5% with each year of incarceration (Carbone Ada et al., 2015). This finding highlights the cumulative risk of TB transmission among prisoners, suggesting that prison was an important place to influence TB transmission. TB in prisons might be caused by imported strains or endogenous reactivation of prisoners (Guerra et al., 2019). Prisoners were mostly from low economic and social status groups, who often had limited access to health care. A previous study reported an outbreak of TB in a US prison housing PLWH, even with a surveillance system, which subsequently led to an increase in TB positive rate in the community (Mclaughlin et al., 2003; Arroyave et al., 2017). highlighting the need for TB control measures in such an environment and the need for multiple interventions to interrupt TB transmission in prisons. A combination of biomedical interventions (early diagnosis, treatment of TB patients, and screening and preventive treatment for LTBI) and structural interventions (reducing overcrowding, improving ventilation and prison conditions) was needed (White et al., 2012). Implement a standardized TB screening program (individuals can be screened at the time of entry and regularly screened after entry to prison, To detect infection) (Margolis et al., 2013), timely isolating of suspected and confirmed infectious TB patients, treatment in patients with active TB, and preventive therapy in patients with LTBI to further reduce the morbidity and mortality (Aguilera et al., 2016), and for close monitoring of prison time longer prisoners, might help reduce TB bacilli in this environmental and community-wide transmission.

This study also showed that the positive rate of LTBI among ethnic minority PLWH was higher than that of Han, probably because most ethnic minority patients came from areas with scarce natural resources, underdeveloped economic, weak primary health care foundation, inadequate institutional mechanisms for TB control, more migrant workers, high mobility, poor living, and housing conditions, and more opportunities for infection from contact with infectious patients, The low awareness rate of TB prevention and control, limited access to other drugs, poor treatment compliance and irregular medication were related to this population. Strengthen health propaganda and education, and popularize knowledge of TB prevention and treatment (Yang et al., 2020). Improved the health awareness of the ethnic minority population, strengthened the health education of TB among the key population, changed the poor living habits, diagnose and treat the disease early, detected the source of infection as early as possible, improved the timeliness of medical treatment, and reduced the chance of transmission (Wang et al., 2019).

This study showed that the positive rate of LTBI in female PLWH was higher than that in male, which was inconsistent with the results of some studies (male had a higher positive rate of LTBI) (White et al., 2001; Baillargeon et al., 2004; Carbone Ada et al., 2015), for example, in Spain: male are higher than female (55.4% vs 25%) (López De Goicoechea-Saiz et al., 2018). Only 10.3% of the subjects were female in this study. Male and female PLWH were from different prison wards. Possibly due to the different prison settings resulting in a higher LTBI rate in female than male, a result that should be considered with caution due to the lack of accuracy in our study as it was a cross-sectional study (López De Goicoechea-Saiz et al., 2018).

The positive rate of LTBI was elevated in PLWH with a contact history with TB patients, and one study had shown a high positive rate of LTBI among TB contacts (29.4%) (Aguilera et al., 2016). A study conducted in Medellin from 2005 to 2006 showed that the positive rate of TST in HHCs of TB patients was 65.9% (del Corral et al., 2009), significantly higher than in the general population. A study in Vietnam showed that the incidence of people in contact with TB patients was 2.5 times higher than in the general population (Fox et al., 2018), and it was well known that some of the risk factors contributed to LTBI in prisons (Rueda et al., 2014) was the high incidence of TB in these settings, the level of overcrowding and close contact between prisoners. Focused on TB screening and preventive treatment of PLWH with a contact history with TB patients.

Our study has the following limitations: First, the median CD4 counts in our study population were high (376) and it was difficult to assess the performance of these tests at low CD4 counts and it was not appropriate to generalize the current results to other PLWH. Moreover, our study was cross-sectional, which makes it difficult to assess the possible association between positive tests and TB progression. Lastly, these populations belonged to prisons in Jiangsu province and may not be fully representative of the general population of PLWH. We, therefore, recommend further studies and follow-up observations in PLWH with CD4 counts below 200, using larger samples and recruiting PLWH on a multicenter scale.

In conclusion, our findings suggest that female, ethnic minorities, history of incarceration, longer duration of incarceration, and contact history of TB patients are risk factors for LTBI among PLWH. Although WHO consolidated guidelines on TB suggested the need for preventive treatment for PLWH without active TB. However, there is still a need to focus on PLWH with the above risk factors for LTBI screening and preventive treatment.

## Data availability statement

The data that support the findings of this study are available on request from the corresponding author. The data are not publicly available due to privacy or ethical restrictions.

## Ethics statement

This study was reviewed and approved by the Ethics Review Board of the Jiangsu Provincial Centre for Disease Control and Prevention. Written informed consent to participate in this study was provided by participants.

## Author contributions

YZ and PL conceived the study, analyzed the data, and drafted the manuscript. DC, KW, HZ, HBY, JY, LD, and WL participated in the study design. HTY and LZ implemented the field investigation. All authors contributed to the study and have read and approved the final manuscript. All authors contributed to the article and approved the submitted version.
